# Cluster-Enhanced Nanopore Sensing of Ovarian Cancer Marker Peptides in Urine

**DOI:** 10.1021/acssensors.3c02207

**Published:** 2024-01-29

**Authors:** Thomas W. Rockett, Mohammed Almahyawi, Madhav L. Ghimire, Aashna Jonnalagadda, Victoria Tagliaferro, Sarah J. Seashols-Williams, Massimo F. Bertino, Gregory A. Caputo, Joseph E. Reiner

**Affiliations:** † Department of Physics, Virginia Commonwealth University, Richmond, Virginia 23284, United States; ‡ King Fahd Medical Research Center, King Abdulaziz University, Jeddah 21589, Saudi Arabia; § Department of Chemistry and Biochemistry, Rowan University, Glassboro, New Jersey 08028, United States; ∥ Department of Forensic Sciences, Virginia Commonwealth University, Richmond, Virginia 23284, United States

**Keywords:** nanopore, cancer, LRG-1, peptides, α-hemolysin, nanoparticles, diagnostics

## Abstract

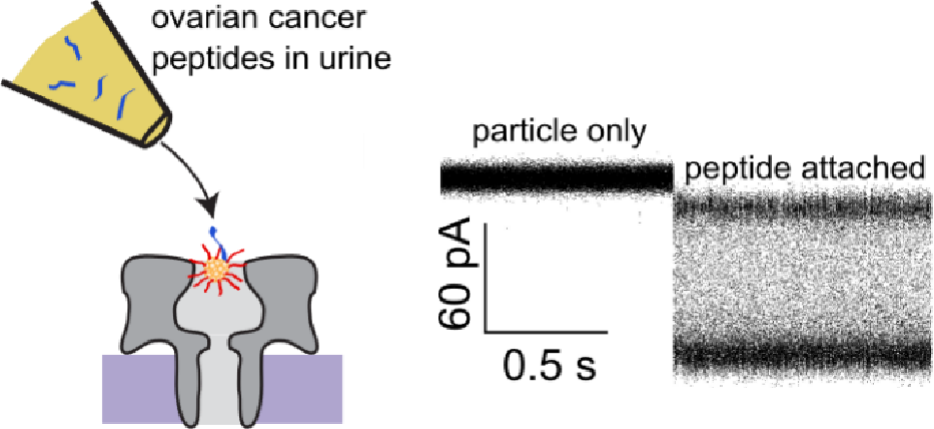

The development of novel methodologies that can detect biomarkers from cancer or other diseases is both a challenge and a need for clinical applications. This partly motivates efforts related to nanopore-based peptide sensing. Recent work has focused on the use of gold nanoparticles for selective detection of cysteine-containing peptides. Specifically, tiopronin-capped gold nanoparticles, trapped in the cis-side of a wild-type α-hemolysin nanopore, provide a suitable anchor for the attachment of cysteine-containing peptides. It was recently shown that the attachment of these peptides onto a nanoparticle yields unique current signatures that can be used to identify the peptide. In this article, we apply this technique to the detection of ovarian cancer marker peptides ranging in length from 8 to 23 amino acid residues. It is found that sequence variability complicates the detection of low-molecular-weight peptides (<10 amino acid residues), but higher-molecular-weight peptides yield complex, high-frequency current fluctuations. These fluctuations are characterized with chi-squared and autocorrelation analyses that yield significantly improved selectivity when compared to traditional open-pore analysis. We demonstrate that the technique is capable of detecting the only two cysteine-containing peptides from LRG-1, an emerging protein biomarker, that are uniquely present in the urine of ovarian cancer patients. We further demonstrate the detection of one of these LRG-1 peptides spiked into a sample of human female urine.

Peptides play a vital role in various biological processes, and their interactions with enzymes and proteins impact critical functions.^1−3^ More specifically, peptides play a significant role in biosignaling, both as primary signaling molecules and as effectors after the signal is received.^4−7^ This makes peptides an important biomarker for various diseases,^8^ which motivates the development of peptide biosensing. Numerous peptide sensing techniques have been explored with many based on either enzyme-linked immunosorbent assay (ELISA) tests^9^ or mass spectrometry (MS).^10,11^ Given the atomic-level accuracy enabled by MS, this approach has become the gold-standard analysis tool for the identification of peptides. Despite the advantages of these techniques, they are still lacking in availability, cost, and sample throughput, thus creating a need for further development of accurate, low-cost, and robust biosensors for the purpose of peptide detection.

Nanopore sensing is one example of a low-cost, high-throughput, and label-free method that has received considerable attention as a possible candidate for peptide and protein detection.^12−14^ This technique has been utilized in small-scale, hand-held devices like the MinION system (Oxford Nanopore Technologies) for DNA sequencing, and this, in part, drives continued efforts to expand the technique to peptide detection. This has led to a variety of successful outcomes in detecting and discriminating select peptides,^15^ but the far more complex problem of sequencing proteins, as compared to DNA, has resulted in a limited number of protein sequencing demonstrations.^16^ Therefore, a need exists to continue to advance nanopore techniques for peptide identification applications.

Improvements to nanopore-based peptide sensing have typically focused on modifications to the pore to yield higher capture rates, longer interrogation times, and/or clearer separation between the signals created by different peptides.^17−21^ Some examples of this include using engineered pores to discriminate between peptides that differ by a single amino acid,^22^ identify post-translational modifications,^23,24^ and characterize enzyme cross talk in the renin–angiotensin system.^25^ In each case, the selectivity and binding of the nanopore can be tuned through site directed mutagenesis (e.g., inserting charged residues in the pore lumen) to enhance the ability of the pore to capture and discriminate between different target peptides. However, given the large number of peptides that can be present in typical samples, (e.g., a tryptic digest of bovine serum albumin (BSA) yields 74 different peptides), nanopore sensors will struggle with peptide discrimination in real-world applications. This issue has been alluded to and addressed through the use of spectral “fingerprinting”,^26^ but the limited resolution of nanopore sensing could be ameliorated by increased detection selectivity, which would reduce the variability in detecting complex peptide mixtures.

Motivated by this need, our group has demonstrated cysteine-selective peptide detection utilizing gold cluster-enhanced nanopores.^27^ By transiently introducing a gold nanoparticle into the cis-side of a wild-type α-hemolysin nanopore, we’ve shown that only peptides containing a single cysteine residue can be captured and analyzed for extended periods. For the case of BSA mentioned above, this reduces the number of detectable peptides from 74 to 13, which is a more manageable number for the nanopore sensor. More generally, it is worth noting that cysteine is the least abundant amino acid in the proteome (<2% of all amino acids in the UniProt TREMBL database are cysteines),^28^ making it an ideal target for reducing peptide mixture complexity. Additionally, we note that accurate diagnostics often requires the ability to simultaneously detect more than one marker.^29^ We believe that cysteine-selective detection provides an unexplored approach to this problem where the sensor does not rely on highly specific transducers (e.g., aptamers) but rather on the gold nanoparticles that enable the detection of a broader population of target peptides (those that contain cysteine residues).

Although nanopore sensing, modified with gold metallic clusters, shows promise for selective detection of cysteine-containing peptides,^27^ it has not yet been applied to the detection of biologically relevant peptides. This account reports our observations of cluster-based nanopore detection of biomarker peptides based on known indicators of ovarian cancers. Specifically, we selected 13 different peptides that each contained a single cysteine residue, making them ideal targets for our selective detection methodology. In addition, each peptide has been shown to be uniquely present in the urine of ovarian cancer patients.^30^

Ovarian cancer remains one of the deadliest forms of cancer (5 year survivability rate of ≈50%)^31^ due in part to difficulties associated with early detection. There are testing methods that include transvaginal ultrasound and CA-125 detection in blood, but a proper diagnosis typically requires general surgery. This motivates the need to develop accurate detection protocols for ovarian cancer biomarkers in bodily fluid samples. Urine presents a compelling option given the ease of acquiring large sample volumes. This motivated our search for peptides that are uniquely present in the urine of ovarian cancer patients.

In this paper, we begin by detecting small cancer marker peptides (<10 residues) and peptides corresponding to proteolytic cleavage products of larger biomarker peptides. These peptides give rise to step-like fluctuations with magnitudes that scale with peptide mass.^27,32^ This shows promise, but the fact that a single data point results from each current step (i.e., one peptide yields one data point) limits the applicability of cluster enhanced nanopore detection. Therefore, we also report on the detection of larger cancer marker peptides (>12 residues), which give rise to so-called “high-frequency” fluctuations.^27^ These fluctuations last several seconds and can be analyzed to extract a variety of metrics that are able to uniquely identify each peptide. This is illustrated here in several instances where we show clear differences between five different cancer marker peptides that are each 16 residues in length, and we also demonstrate clear discrimination between differently sized peptides. We demonstrate that the cluster-enhanced nanopore sensor can detect and differentiate the only two cysteine-containing peptides originating from leucine-rich α-2 glycoprotein 1 (LRG-1) protein biomarkers that have been shown to be present in the urine of ovarian cancer patients.^30^ This is important because LRG-1 is becoming a highly sought after target for disease monitoring,^33^ and this technique could be used as a detector. Finally, we report initial results on the detection of one of the LRG-1 peptides spiked into human female urine, which provide a proof of concept for our technique to be developed into a viable sensor with clinical applications.

## Results and Discussion

The physicochemical properties (sequence, length, mass, charge at pH 8.0) for each of the peptides studied herein can be found in Table 1, Section 1 of the Supporting Information. We analyzed 13 peptides and 5 fragments that were selected from a database of peptides that uniquely present in the urine of ovarian cancer patients.^30^ These peptides contain a single cysteine residue, which is necessary to facilitate attachment to the pore-bound gold nanoparticle.^27^ The nanopore opening and cluster are on the order of 2 nm,^34^ so peptides were selected that range in length between *n* = 8 and 23 residues (<10 nm fully extended). The five peptide fragments are sequences that would result from trypsin or chymotrypsin digestion from some of the peptides listed in the upper portion of the table. Table 2, Section 1 of the Supporting Information lists each of these cancer biomarker peptides along with the corresponding full protein from which each peptide originates. We also list the full length of each protein, the location of the peptide within the full protein sequence, and the three residues before and after each peptide within the full protein sequence.

**Figure 1:**
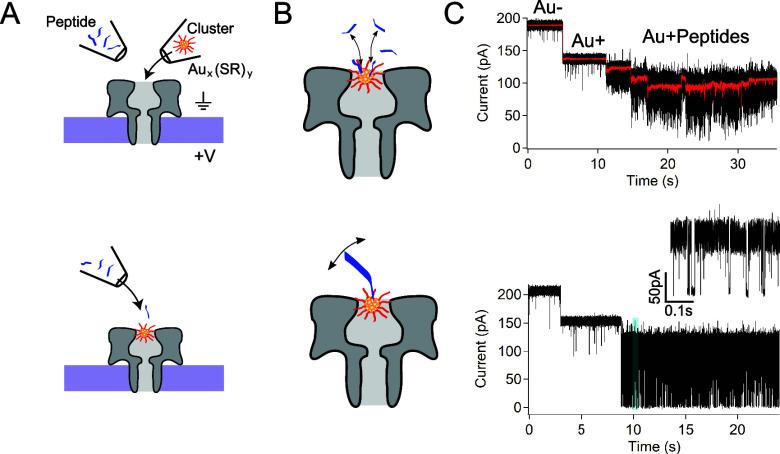
Cluster-enhanced cysteine-selective nanopore sensing. (A, top) The multistep process begins with a peptide-containing and cluster-containing nanopipette tip positioned in the vicinity of an isolated nanopore. Clusters are ejected into the pore until one is captured. (A, bottom) The cluster tip is removed, and peptides are ejected toward the cluster-containing pore. (B) Two types of fluctuations ensue. (Top) For smaller peptides, the clusters attach or detach on a seconds time scale, whereas (bottom) larger peptides show a single peptide attachment with subsequent high-frequency fluctuations. (C) Typical current traces for these two event types show (top) attachment of smaller peptides or (bottom) a single large peptide. The smaller peptide trace (black) is overlaid with a 100 Hz low-pass filtered signal (red) that clearly demonstrates the discrete attachment steps. In both current traces, transitions from the open-pore state (*i*_0_ ∼200 pA) to the cluster occupied state (*i*_c_ ∼140 pA) precede peptide detection. The lower trace shows an inset corresponding to the blue box around *t* = 10 s that illustrates the complex nature of the resulting fluctuations.

The principle of operation for the cluster-based nanopore detector has been previously described.^27,32^Figure 1 provides an illustration of the technique along with typical peptide-induced current traces. Briefly, a single, wild-type α-hemolysin pore is inserted into an artificial lipid bilayer membrane, and two micropipette tips are positioned in proximity to the pore. Backing pressure is applied to the tip containing preformed gold nanoparticles until a single gold cluster is captured in the pore. After this, the gold-containing tip is removed, and pressure is applied to the second tip containing the peptide of interest. This leads to peptide attachment to the gold cluster through either direct addition or ligand exchange.^32^ When small peptides (5–10 residues) are introduced near the cluster, we see a number of downward current steps in the filtered signal (red trace, Figure 1C) corresponding to the addition of multiple subsequent peptides. Larger peptides (>10 residues) ejected onto the cluster typically yield a single attachment and long-lived fluctuating states.^27^

**Figure 2:**
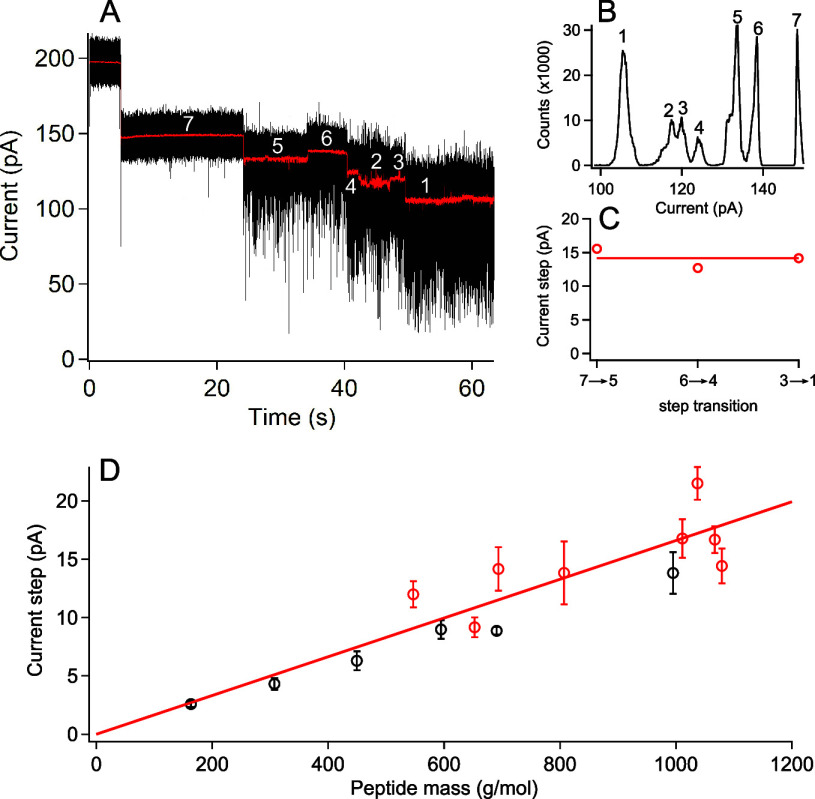
Cancer marker peptides exhibit low-frequency stepwise fluctuations that correlate with peptide mass. (A) Sample current trace for P9C6 peptide. The raw trace (black) with the overlaid filtered data (100 Hz low pass, red) that exhibits clear steps between the numbered states. (B) A current histogram of the filtered data yields seven distinct peaks, which are labeled with the white numbers in panel A. The transitions from states 7 to 5, 6 to 3, and 4 to 1 yield three current steps plotted in panel C. (D) A summary of all cancer marker peptides (red circles) yields a similar but more scattered trend than previously analyzed control peptides (black circles) (see Figure 4 in ref (27)). Each red data point in panel D corresponds to the mean and standard deviation measured from the following number of steps and pores: P8C5 = (6, 2), P9C6 = (6, 1), P9C9 = (3, 1), P9C6FC = (8, 3), P9C9FC = (11, 2), P16C1FT = (6, 2), P16C9FT = (8, 1), and P16C15FT = (9, 2). The cancer marker peptide data in panel D are least squares fit with a linear function (solid red line) and fixed origin to a slope of 16.6 ± 0.5 fA/(g/mol), which is slightly larger than the least squares fit to the previously analyzed synthetic peptides (black circles, fit not shown) (13.4 ± 0.4 fA/(g/mol)).^27^

Figure 2A highlights a typical current trace and corresponding all-points histogram (Figure 2B) of the filtered data (red trace, Figure 2A) from a so-called “low-frequency” peptide (P9C6; see Section 1, Table 1 in the Supporting Information). Numerous attachments of this peptide yield current steps whose magnitude are measured from the peak positions in Figure 2B. Similar analysis (see Section 2, Figure 1 in the Supporting Information) for all the small peptides and fragments (P8C5, P9C6, P9C9, P9C6FC, P9C9FC, P16C1FT, P16C9FT, and P16C15FT) yields the mean current step associated with each of these peptides. These mean values are reported as red circles in Figure 2D, and these data show a generally linear dependence between peptide mass and current step magnitude. This is consistent with our previous study that analyzed current step magnitudes for a number of model peptides and ligands (black circles reproduced from ref (27)).

Although the trendline shows clear agreement between the model and cancer marker peptides, the scatter in the cancer marker peptides suggests that relying on the current step alone may not provide sufficient discrimination between different peptide sequences for sensing purposes. The increased scatter for the cancer marker peptides is not surprising given the random nature of the sequences studied herein as compared to the previously analyzed peptide sequences.^27^ These stepwise fluctuations are also limited because each step only yields two bits of information: the current step magnitude, which scales with the peptide mass,^35^ and the duration of each current substate, which scales with peptide concentration. Therefore, these low-frequency fluctuations may not be useful for unambiguously identifying the peptide. Nevertheless, this nanoparticle approach could still prove useful given the cysteine-selective detection provided by the cluster, but it is most likely that standard open-pore analysis will be more effective at detecting peptides in the smaller size range (*n* ≤ 10).

The advantage of our nanoparticle-based approach becomes more obvious for larger peptides that give rise to so-called “high-frequency” fluctuations. We have previously shown^27^ that the attachment of larger peptides yields equal magnitude downward current steps, which suggests that individual peptides can be bound to the cluster, which are overlaid with high-frequency multistate fluctuations. This enables the analysis of individual peptides over extended periods, which gives a more complete picture of the peptide due to its longer interaction time with the cluster-modified nanopore. Motivated by this, we begin by analyzing four different-sized peptides (P13C1, P16C1, P20C2, and P23C1) with the cysteine residue at or near the N-terminus of the sequence. Each of these peptides gives rise to long-lived high-frequency fluctuations when analyzed with the nanoparticle-occupied pore. We compare this to more traditional open-pore analysis and show that the nanoparticle approach yields important advantages.

**Figure 3:**
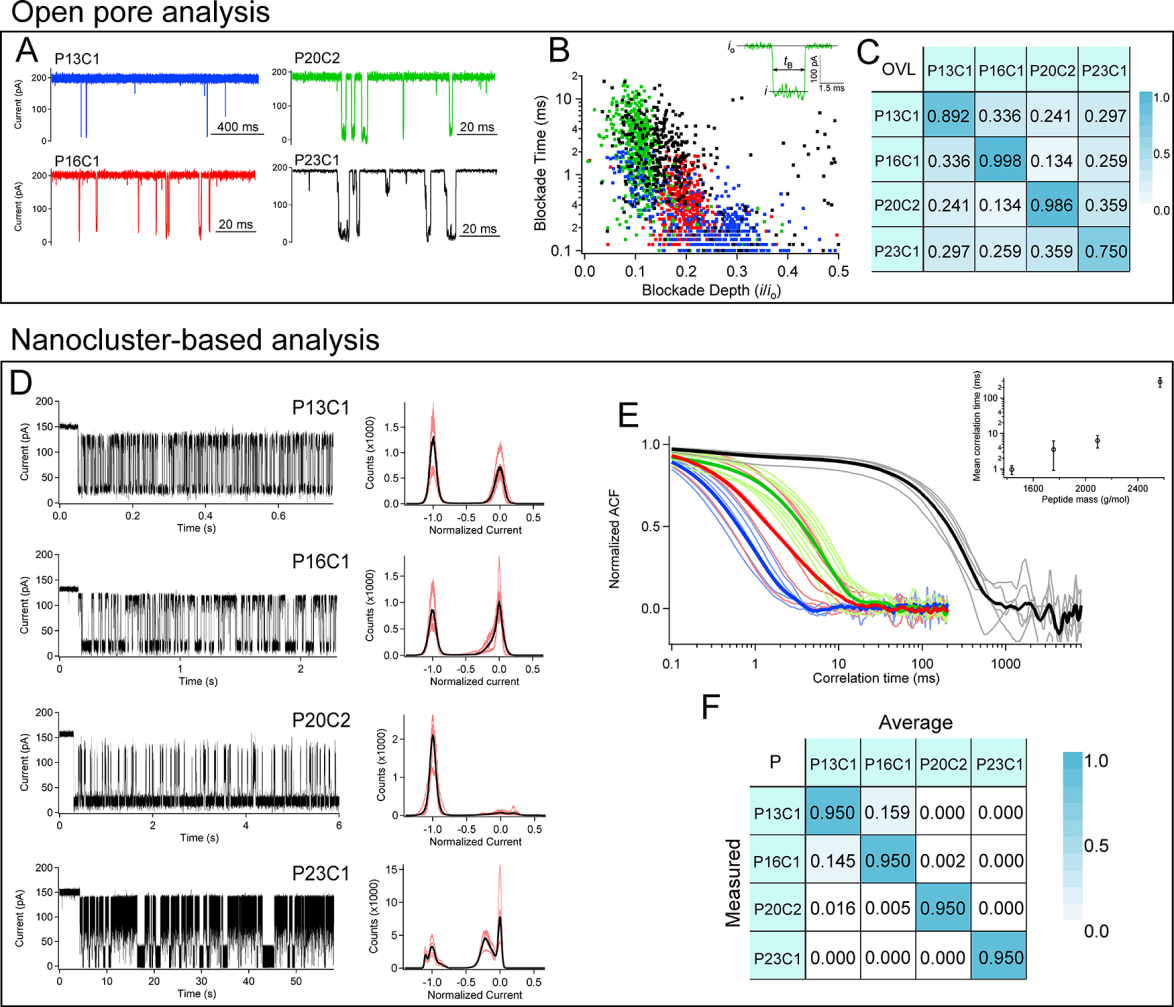
Cluster-based high-frequency fluctuation analysis of differently sized cancer marker peptides yields a more accurate discrimination than open-pore analysis. Panels A–C correspond to open-pore analysis. Panels D–F correspond to the cluster-based analysis. (A) Sample current traces of open-pore analysis show various current blockades corresponding to the different peptides analyzed. (B) Scatter plot of the blockade time and normalized blockade depth for 500 blockade events from each peptide. (B, inset) A sample current blockade with the open-pore current (*i*_0_), blockade current (*i*), and blockade time (*t*_B_). (C) Overlap percentage matrix for peptide pairs shows ≈25% overlap between each peptide. The nonunity values along the diagonal result from the fact that the overlap calculation was confined to the axis range shown (0 < *t*_B_ < 20 ms and 0 < *i*/*i*_0_ < 0.5) and not all blockade events fall within these boundaries. (D) Sample traces and corresponding all-points histograms from each peptide attached to a pore-bound cluster. Black curves in the histograms correspond to the average histogram from a minimum of five cluster events. Individual cluster events shown as pink traces. (E) Normalized autocorrelation for each peptide (color coding matches peptide identification from part A, fine curves are individual events, and the bold lines show each average). (E, inset) Least squares fits of the averaged ACF with single exponential offset functions (*A*exp(−(*t – t*_0_)/τ_mean_) show that the mean correlation times scale exponentially with the peptide mass. (F) Probability matrix for identifying a measured peptide (down a column) when compared against the averaged histogram and correlation time (along rows) (see data analysis in Section 3 of the Supporting Information). For example, the probability of detecting a P16C1 peptide and identifying it as a P13C1 peptide is 14.5%.

Figure 3A shows typical current traces in the open-pore configuration with various current blockade signatures. The two smaller peptides yield short-lived downward-going spikes, and the larger peptides show longer-lived blockades with a multistate structure. The charge-neutral peptide (P13C1) exhibits a far lower on-rate to the pore and thus required a different time scale to report several blockade events. The current blockades are all consistent with previous work that showed that larger molecules spend more time in the pore.^36^ This most likely results from increased enthalpic interactions between the peptide and nanopore.^37^ Open-pore analysis typically compares the blockade depth (*i*/*i*_0_) and blockade durations (*t*_b_) between different peptides as shown in Figure 3B. Here we report distributions of 500 blockade events for each peptide, and we use the overlap percentage (Figure 3C, overlap calculation described in Section 3 of the Supporting Information) to estimate the likelihood of a specific blockade being incorrectly assigned to a given peptide. For example, there is a 33.6% chance of identifying a P13C1 blockade as originating from a P16C1 peptide and vice versa. The diagonal elements are less than one because the overlap was calculated in the range shown in Figure 3B (0 < *i*/*i*_0_ < 0.5 and 0.1 ms < *t*_b_ < 20 ms), and some events fall outside this range (e.g., 89.2% of the P13C1 events are within the range shown in Figure 3B). As can be seen from the off-diagonal elements, there is a considerable degree of overlap for peptides that differ in size over the reported range (i.e., 1400–2600 g/mol), and this will complicate the open-pore sensor’s ability to correctly identify peptides in multicomponent mixtures.

The nanoparticle-based detection improves the selectivity of the nanopore in part because each molecule remains on the sensor for extended periods. Figure 3D shows typical current traces where a gold nanoparticle is captured by the pore and a peptide is subsequently attached to the cluster. High-frequency fluctuations ensue, which yielded complete details of the current distribution. Interestingly, the smaller peptides (P13C1 and P16C1) exhibit two-state fluctuations, which have been described previously,^27^ and larger peptides (P20C2 and P23C1) exhibit multistate fluctuations. These multistate fluctuations are suggestive of the peptide folding into different conformational states while bound to the nanoparticle, and a previous study explored a similar behavior in a different system.^38^ Although free solution tryptophan fluorescence studies of the P23C1 peptide and circular dichroism spectra of both the P20C2 and P23C1 peptides show little evidence of the secondary structure or aggregation (see Section 4, Figures S2 and S3, in the Supporting Information), it is possible that the cluster-pore environment and binding of the cysteine to the cluster lead to structural forms that are observable with the nanopore sensor that are not present in free solution. In any event, analysis of these multistate fluctuations and transition frequencies between them is beyond the scope of the present manuscript, but they may serve to further improve discrimination between larger peptides. Here, we use both the current distribution and the mean correlation times to more accurately identify each peptide in this collection.

To analyze nanoparticle induced fluctuations, we used a chi-squared-based approach that calculates the probability of incorrectly identifying one peptide as another from the current histograms. We then calculated the autocorrelation function of each current trace to extract the mean and standard deviation of the correlation times for each peptide. From these results, we calculated the probability of measuring a given peptide and identifying it as a different peptide and reported that in the so-called *P*-matrix (probability) shown in Figure 3F. More complete details describing these calculations can be found in the data analysis section of the Supporting Information. As an example of the meaning of the elements of the *P*-matrix, we find a 14.5% probability of measuring a P16C1 peptide and incorrectly identifying it as a P13C1 peptide. From Figure 3F, it is clear that the nanoparticle approach is more effective at correctly identifying these differently sized cancer marker peptides than the open-pore analysis. It is also worth reiterating that the cluster-based approach is only sensitive to cysteine-containing peptides, which will greatly reduce the complexity of measuring peptide mixtures.

**Figure 4:**
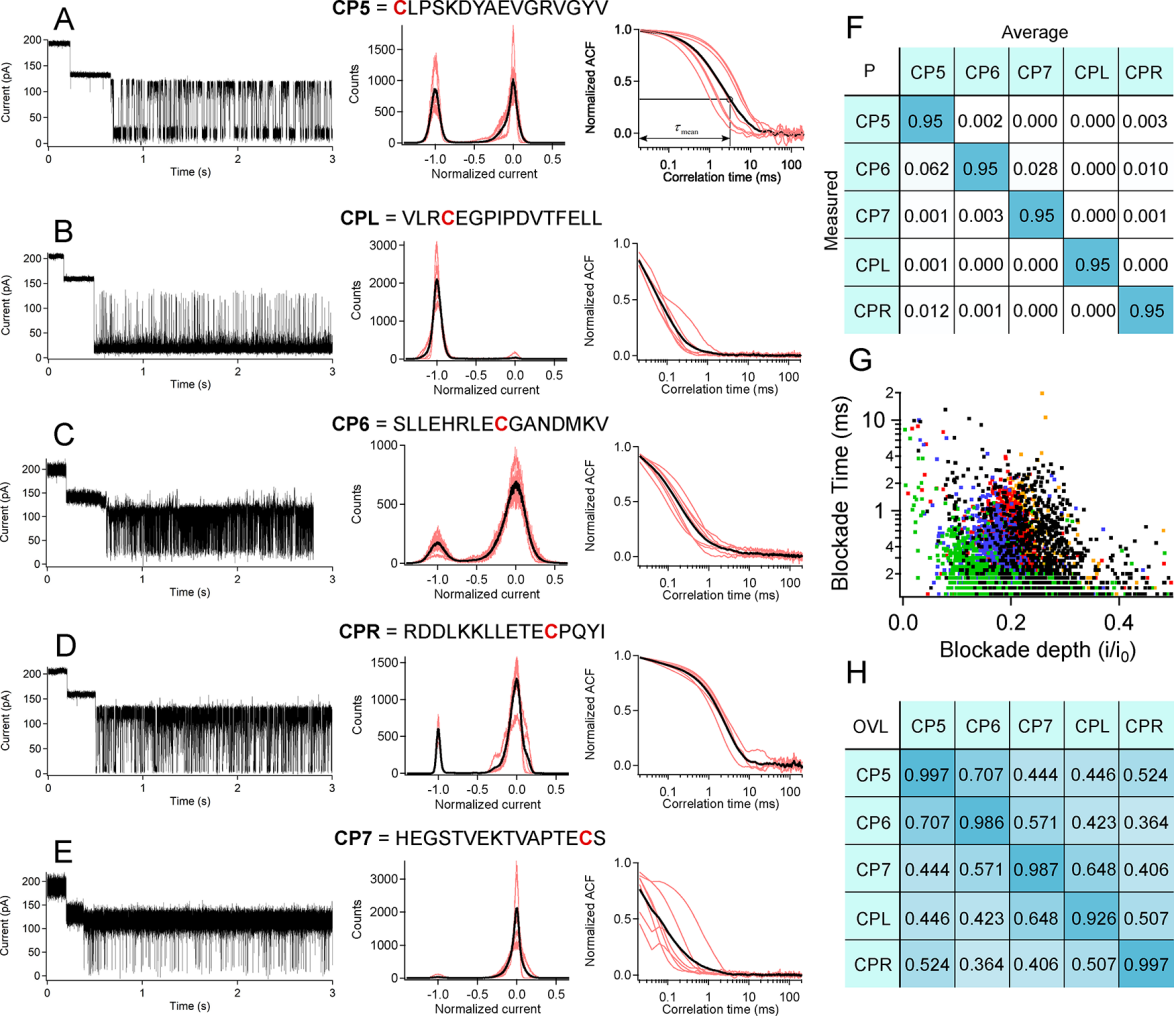
High-frequency fluctuation results from similar-mass 16-mer cancer marker peptides show the superiority of the gold cluster approach compared to open-pore analysis for discriminating between peptides. The single cysteine residue is positioned at different positions within each peptide sequence. (A–E, left) Cluster-based sample current traces for each peptide type with (A–E, middle) corresponding all-points histograms of the peptide-induced fluctuations. (A–E, right) Corresponding autocorrelation functions for each trace averaged over a minimum of five different cluster captures yield distinct correlation time fluctuations. (F) The probability of incorrectly identifying a measured peptide (along a column) against the average histogram and correlation time (along the row) is well below 5% and near zero in almost all cases. The only significant exception is the 6.2% probability of identifying a P16C9 peptide as a P16C1 peptide. (G, H) Open-pore analysis shows a far greater overlap in the scatter distribution as expected for peptides with the same number of residues.

To further demonstrate the strength of the gold-cluster method, we analyzed similar-sized peptides where open-pore fluctuations usually result in more ambiguous interpretations and thus higher uncertainty in distinguishing between different molecules. Figure 4 compares the nanoparticle and open-pore analyses for peptides P16C1, P16C4, P16C9, P16C12, and P16C15. Each peptide is 16 residues in length (and therefore approximately equal in mass (±106 amu)), but each has a different sequence, and the cysteine residue is located at different positions along each sequence. Figure 4A–E shows sample current traces of the cluster-based detection of each peptide along with corresponding current histograms and normalized autocorrelation functions. From each current distribution and autocorrelation function, we again calculate the detection probability matrix (*P*-matrix, Figure 3F). Most of the off-diagonal elements are less than 1%, with the largest probability (6.2%) corresponding to incorrectly identifying a measured P16C9 peptide as P16C1. This is in striking contrast to the overlap probabilities of the open-pore blockades shown in Figure 4G,H where most peptide-pairs show an approximately 50% overlap. We note a higher degree of variance in the autocorrelation functions for P16C1 and P16C15. This is most likely related to the variability in the nanoparticle composition and/or differences in the binding location of the peptide onto the particle. To help the reader better visualize these differences, we present current traces for each of the seven events for P16C1 and P16C15 in Figures S4 and S5 in Section 5 of the Supporting Information.

As already mentioned, the cluster-based approach provides a considerable degree of selectivity because the gold clusters only yield signal from cysteine-containing peptides. This is amplified by the fact that cysteine residues are a naturally occurring amino acid with among the lowest abundance in proteins,^28,39^ adding an additional layer of selectivity to the method. This will greatly reduce the complexity of detecting the numerous peptides that result from the proteolytic digestion of various proteins. As an important example of this for detecting cancer biomarker peptides, we explore the detection of peptides resulting from the digestion of human leucine-rich α-2 glycoprotein 1 (LRG-1) proteins (GenBank: KAI4039707.1).^33^

**Figure 5:**
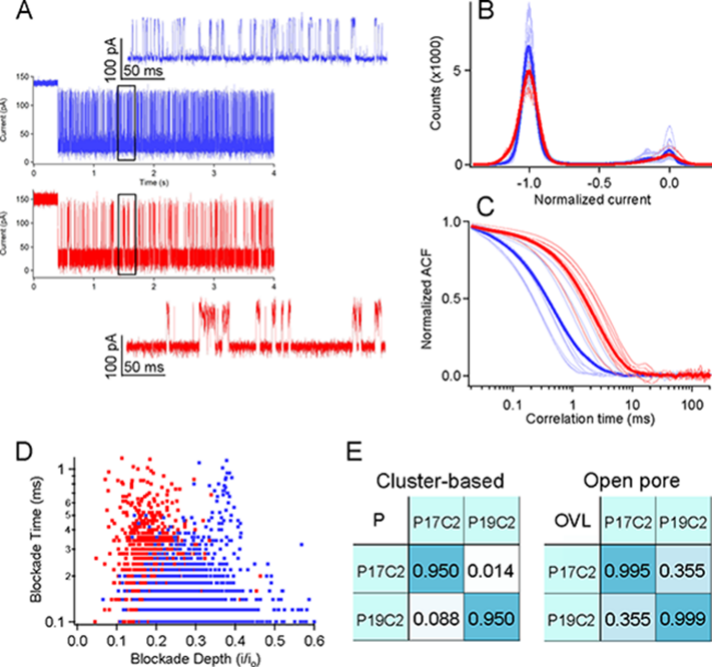
Only two cysteine-containing peptides from the LRG-1 protein that present in the urine of ovarian cancer patients (P17C2 (blue) and P19C2 (red)) yield clearly detectable fluctuations in the nanoparticle-based sensor. (A) Typical current traces show a single gold nanoparticle from *t* = 0 to 0.4 s when a single peptide is captured. This yields clear fluctuations, and a zoomed-in view between *t* = 1.4 and 1.7 s (highlighted by black rectangles) shows clearly discernible differences in the kinetics. The larger peptide (P19C2) remains in the high current state for longer periods of time. (B) Averaged all-points histograms of the current fluctuations (bold traces) along with the eight different distributions for each peptide from which the averages are calculated. (C) Autocorrelation analysis shows differences in the kinetics for each peptide, and least squares fits to each correlation trace yield mean correlation times for the two peptide types: τ_P17C2_ = 0.77 ± 0.56 ms and τ_P19C2_ = 2.7 ± 0.9 ms. (D) Open-pore scatter plots show a high degree of overlap. (E) Comparison of the separation probability matrix for cluster analysis and the open overlap distributions show greatly enhanced selectivity for cluster-based analysis (<10% for cluster analysis and 36% for open-pore analysis).

LRG-1 has received increasing attention for its role in many diseases. It has been directly connected with the onset and progression of eye, kidney, lung, heart, and various inflammatory diseases. In addition, LRG-1 has been associated with a wide variety of cancers, including ovarian cancer.^33^ For the case of ovarian cancer, it was shown that LRG-1 peptides were only found in the urine of six ovarian cancer patients, whereas only one LRG-1 peptide (GKDLLLPQPDLRY) appeared in one of six healthy (control) patients.^30^ This suggests that detecting LRG-1 peptides, in addition to the simultaneous detection of other peptide markers, like those described herein, could serve as an effective method for identifying the onset of ovarian cancer. We demonstrate here that our nanoparticle-based nanopore sensor could detect the presence of LRG-1 protein fragments. LRG-1 has been reported as the most abundant protein biomarker in the exosomes found in the urine of ovarian cancer patients^40^ and may be linked to several other conditions (appendicitis^41^ and renal failure^42^). Additionally, there are 90 different peptides originating from LRG-1 that are uniquely present in the urine of ovarian cancer patients.^30^Figure 5 shows typical current traces and corresponding analysis of the only two cysteine-containing peptides from this collection of 90 (P17C2 and P19C2). The nanoparticle-based analysis shows that both peptides are detectable and yield clear differences in the high-frequency fluctuating current traces. The chi-square and correlation time analysis yields a less than 10% likelihood of incorrectly identifying one for the other. We note that there is a 35.5% overlap from the open-pore blockade analysis of these two peptides, but the nanoparticle approach reduces the complexity of detecting LRG-1 because it is sensitive to only these two peptides (of the 90 from LRG-1). This reduction in detectable peptides should simplify signal analysis requirements when applied to more complex clinically isolated samples.

**Figure 6:**
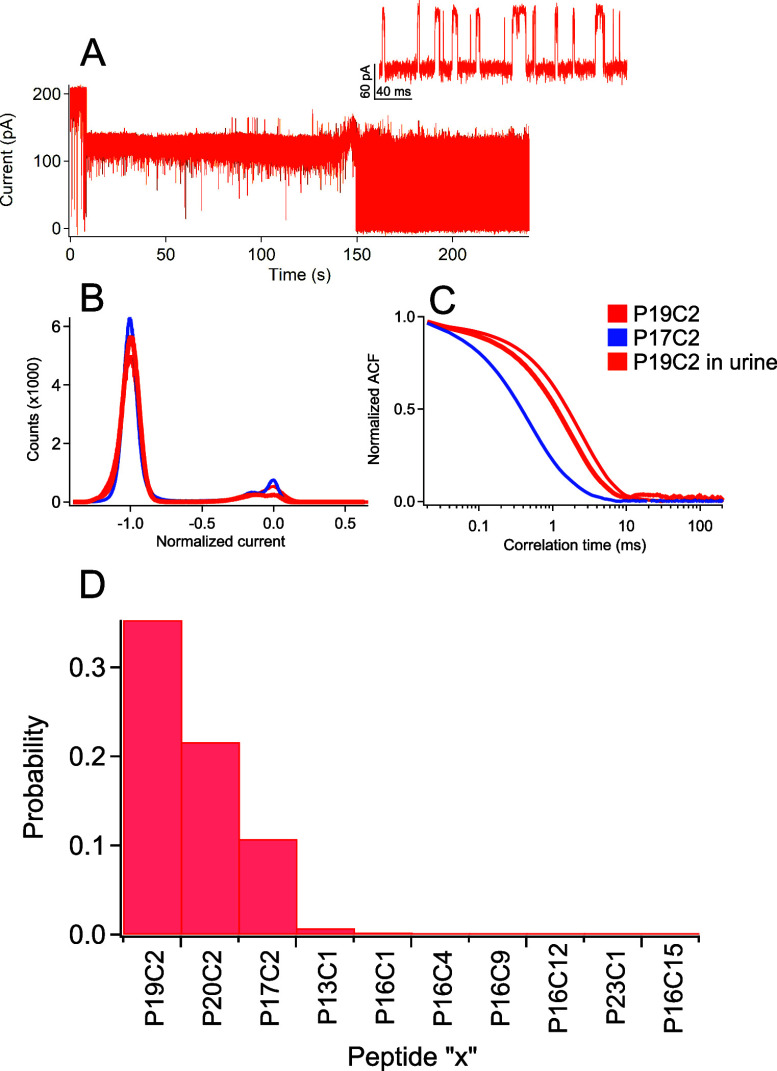
P19C2 detection in urine demonstrates the feasibility of working in bodily fluids. (A) The membrane and pore remain stable under exposure to the urine and yield capture of a P19C2 peptide at 150 s. The inset shows a zoomed-in view of the post peptide-capture trace. (B) Comparing the normalized current histogram for this particular peptide event to the averaged histograms for P17C2 (blue) and P19C2 (red) in the clean buffer (see Figure 5) shows close agreement with both. (C) Autocorrelation analysis also shows close agreement between the P19C2 urine and clean buffer samples. (D) Quantitative analysis shows that at 95% confidence interval, there are a 35.3% chance of identifying this event as a P19C2 peptide and a 10.7% chance of identifying this event as a P17C2 peptide. A summary comparing this event against all the other measured peptides (peptide “*x*” in the clean buffer) from Figures 3 and 4 shows excellent selectivity and the feasibility of urine-based detection.

If the nanopore sensor is to be a viable detector of these ovarian cancer marker peptides, it should be compatible with samples derived from human urine. There have been a few reports of incorporating bodily fluids into nanopore detection schemes,^43−46^ but the viability of nanopore sensors in bodily fluids depends on the relative concentration of membrane partitioning proteins, lipids, and peptides contained in the fluid.^46^ This means blood, serum, and saliva are not ideal for nanopore detection, but peptide detection in urine may be possible. To demonstrate this, we spiked LRG-1 peptide P19C2 into human female urine and introduced this mixture onto the cluster-occupied pore. Figure 6A shows a sample trace with little evidence of damage to the nanopore or membrane support and the capture of a peptide that exhibits fluctuations similar to those observed for P19C2 peptides in the clean buffer (Figure 5A). We compared both the normalized histogram distributions (Figure 6B) and autocorrelation functions (Figure 6C) between the urine-spiked peptide and the clean-buffered peptides and found considerable overlap with the matching P19C2 peptide. Finally, we calculated the probability of identifying the urine-based peptide fluctuations with all the other “high-frequency” peptides studied herein and found that the most likely probability was that the urine-based fluctuations originate from the P19C2 peptide as expected. The only other peptides that showed significant overlap probabilities with the urine-based peptide were P20C2 (22%) and P17C2 (11%). The P17C2 result is nearly identical to the overlap observed in the buffer, and the strong overlap with P20C2 most likely results from the similar peptide mass between this and P19C2. Regardless, the results in Figure 6 highlight the feasibility of the nanoparticle detection scheme in urine, further motivating this approach.

It is worth noting that the peptide concentration used in the P19C2 urine measurements is too high for clinical interests (250 μM) given that LRG-1 protein concentrations in urine have been reported to be at the 2 nM level.^47^ However, here, we are interested only in demonstrating that the ensuing fluctuations associated with peptide capture from urine are consistent with those observed in the model system using the buffer as the solvent. A more advanced protocol that will most likely require concentrating steps (e.g., affinity capture/enrichment steps) and premixing of the gold clusters in the urine/peptide solution followed by cluster capture in the pore will be required to move to lower concentration thresholds. Additionally, we note that implementing nanoparticle-based detection will require sample preparation (e.g., size exclusion columns, protease enzymes, reducing agents) to address interfering effects (e.g., cysteine oxidation, disulfide bond formation, nonspecific binding), but this is to be expected when analyzing complex bodily fluid samples. These efforts are beyond the scope of the present work and will be the focus of a later study.

## Conclusions

Nanopore sensing is becoming a viable, low-cost, and portable technique for single-molecule detection and analysis. In particular, there is growing interest in using nanopores for peptide detection. Typical measurements usually result in a large number of short-lived current blockades that yield limited bits of information (e.g., magnitude, duration, and standard deviation) that can be used to identify the peptide. To improve selectivity, there has been a growing interest in exploring more sophisticated data analysis methods,^48^ but there is still a need to improve the quantity and quality of data extracted from each peptide’s interaction with the pore.

In this article, we describe the application of a nanoparticle-based approach for cysteine-selective peptide detection, and we apply it to the detection of a number of ovarian cancer marker peptides. For smaller peptides (<10 amino acid residues), we found a general trend between the current step magnitude and the peptide mass, but the limited data (one bit per peptide) and overall spread, which most likely results from sequence variability, call into question the efficacy of the nanoparticle technique for distinguishing between shorter peptides. However, for larger peptides, the nanoparticle enables long interrogation times for single peptides, and this yields far better discrimination when compared to more traditional open-pore analysis methods. Importantly, the nanoparticle approach was applied to the analysis of the only two cysteine-containing peptides that result from the highly sought LRG-1 protein biomarker in the ovarian cancer peptide library. Additionally, one of these peptides was detected in female human urine. These measurements demonstrated clear discrimination between these two peptides, which suggests that the nanoparticle-based approach applied to a parallel detection setup (e.g., MinION from Oxford Nanopore Technologies) could provide a significant advancement for peptide detection and analysis.

## Methodology

### Materials

1,2-Diphytanoyl-*sn*-glycero-3-phosphocholine (DPhyPC) lipid was purchased from Avanti Polar Lipids (Alabaster, AL, USA). Alpha toxin from *Staphylococcus aureus* was purchased from IBT Bioservices (Rockville, MD, USA). Teflon supports for membrane formation were purchased from Goodfellow USA Corp. (Coraopolis, PA, USA). Holes (50 μm diameter) were formed in the Teflon sheets with laser drilling at Potomac Photonics Inc. (Halethorpe, MD, USA). Borosilicate glass capillaries (with filament) were purchased from Sutter Instruments (Novato, CA, USA). Potassium tetrachloroaurate (III) hydrate, Tris, tiopronin, potassium chloride, hexadecane, citric acid, and potassium hydroxide were purchased from Sigma-Aldrich (St. Louis, MO, USA). The borate-*tert*-butylamine complex was purchased from Alfa Aesar (Ward Hill, MA, USA). *n*-Pentane and methanol were purchased from Fisher Scientific (Washington, DC, USA). All peptides were purchased from GenScript (Piscataway, NJ, USA). All chemicals were used as received without further purification. Urine was collected from a female donor using informed consent, in accordance with the approved Institutional Review Board Human Subjects Research Protocol (HM20002931). Urine was deposited into a sterile collection cup supplied to the donor and returned on ice within 24 h before aliquoting into 1 mL volumes and freezing at −80 C until use.

### Fluorescence Spectroscopy

Samples were prepared in PBS and adjusted to pH 8.0. Samples contained varying concentrations of peptide as denoted in the Supporting Information figures. Independent samples were prepared for each concentration. Fluorescence spectra were collected on a JY Fluoromax 4 with 2.5 nm slit widths. Fluorescence emission spectra from the tryptophan residues in the peptide were measured using 280 nm excitation wavelength, and emission was collected over the range of 300–400 nm. Background spectra from samples containing no peptide were subtracted from the sample spectra. Spectra were normalized such that the highest intensity was equal to 1.0. This calculation was also used to determine λ_max_, the wavelength of highest intensity.

### Circular Dichroism Spectroscopy

Circular dichroism (CD) samples were prepared in PBS or in PBS supplemented with 3 M KCl or 5 M GuHCl. Samples contained 20 μM peptide. CD spectra were collected on a JASCO CD Spectropolarimeter over the range of 190–260 nm, and each spectrum was an average of 64 scans. Background spectra from samples containing no peptide were subtracted from the sample spectra.

### Nanoparticle Synthesis

Nanoparticles were synthesized via reduction of gold salt (KAuCl_4_) in the presence of a reducing agent as well as thiolated ligands, which in this case are the borane *tert*-butylamine complex (BTBC) and tiopronin (TP), respectively. All three elements were separately dissolved to 2.5 mM in methanol and combined in a 2:2:1 molar ratio as follows. First, 700 μL each of gold salt and ligand solutions was mixed and shaken vigorously for 30 s. Then, 350 μL of BTBC was added, and the resulting solution was vortexed for 30 s, after which this solution was sonicated for 30 min. During this time, the solution turned from a clear-yellow to a darker amber color, indicating the synthesis of the gold nanoparticles. We then dried the suspension under a fume hood for ca. 24 h, and the particles were resuspended in ultrapure water (18.2 MΩ·cm) and stored in a 4 °C refrigerator until used. According to previous work,^27,49^ this synthesis produces gold nanoparticles with a significant proportion of particles in the 2 nm range. No further size exclusion was necessary because the α-hemolysin nanopore serves as a sufficient filter.^34^

### Nanopore Sensing

There are numerous reviews describing the general principles of the nanopore sensing methodology.^50^ Our approach has been described previously,^27,32^ and a brief review of this methodology follows. A lipid bilayer is formed across a 50 μm hole in a 20 μm thick Teflon (PTFE) partition with DPhyPC dissolved in hexadecane at a 10 mg/mL concentration. The partition was pretreated with 2 μL of a prepaint mixture consisting of 1 mg/mL DPhyPC dissolved in pentane. Following the formation of a membrane, a single α-hemolysin channel was inserted into the membrane via the so-called tip insertion method. The pore insertion was confirmed with a step-change in current corresponding to the conductance of a single channel, and proper orientation could also be confirmed by examining the current rectification and comparing against known values.^51^ All nanopore measurements were carried out in an aqueous buffer (3 M KCL, 10 mM Tris, pH = 8.0) except for the urine-based measurements. For these measurements, urine was centrifuged for 5 min at 10,000 rpm to reduce cellular and other organic debris from clogging tips. Urine solvent was extracted from near the top of the centrifuge tube following centrifugation, and the peptide was dissolved into this solvent before being added to the micropipette tip.

A 1:4 by volume nanoparticle/electrolyte solution was loaded into a borosilicate capillary (OD = 1.0 mm and ID = 0.78 mm) formed into a micropipette tip using preset program #11 on the P-2000 puller (heat = 350, Fil = 4, Vel = 30, Del = 200) (Sutter Instruments, Novato, CA) with a final ID of 1 to 2 μm. This nanoparticle-filled micropipette was positioned ca. 50 μm above the membrane and ca. 20 μm off the edge of the membrane on the cis-side of the pore (measured via the MPC-200 controller (Sutter)). Unless otherwise stated, a 70 mV transmembrane potential was applied (Axopatch 200B, Molecular Devices, San Jose, CA) with appropriate polarity (ground held fixed on the cis-side of the pore), and a backing pressure of approximately 15 hPa was applied through the tip to eject particles (Femtojet, Eppendorf, Hauppauge, NY). When a cluster enters the pore, a noticeable drop in current occurs. Single particle captures occur because the nanoparticles are anionic in the pH 8 buffer,^52^ making them repulsive at the length scale of the nanopore opening.

To attach peptides to the pore-bound nanoparticle, a second tip was formed in the laser-puller and filled with the peptide of choice. All peptide concentrations in the tips are 500 μM unless otherwise stated. The second tip was positioned an equal distance from the membrane on the opposite side of the membrane with respect to the nanoparticle tip. Once a cluster entered the pore, the pressure to the nanoparticle tip was reduced to zero, and after approximately 10 s, the pressure in the peptide containing tip was increased to ca. 15 hPa. After some time, the peptide attached to the pore-bound cluster, and the current was recorded for extended periods (>10 s).

### Data Processing

Current signals were digitized and collected at a sampling rate of 50 kHz (Digidata 1550B, Molecular Devices) with a four-pole low-pass Bessel filter set to 10 kHz. Current traces were recorded with the pCLAMP 10.7 software (Molecular Devices). Further analysis (i.e., histograms, digital filtering, and multipeak fitting) was performed with IGOR 6.37 (Wavemetrics, Portland, OR). Current signal traces were reported with either the 10 kHz filter or a postprocessed 100 Hz digital filter using the IGOR software (four-pole, infinite impulse response filter). Current steps for Figure 2 were calculated from least squares Gaussian fits to histogram distributions as needed. All plots were generated by using the IGOR software.

## Supporting Information Available

Summary of peptides studied, low-frequency peptide fluctuations analysis, detailed descriptions of the data analysis, peptide CD and fluorescence spectral analysis, and current traces for P16C1 and P16C15 (PDF)

## ABBREVIATIONS

LRG-1: leucine-rich α-2 glycoprotein 1
ACF: autocorrelation function
