# Supplementary material for: Cluster-Enhanced Nanopore Sensing of Ovarian Cancer Marker Peptides in Urine
Source: ACS Sens. 2024 Jan 29;9(2):860–9. doi: 10.1021/acssensors.3c02207 (PMC10897939; doi:10.1021/acssensors.3c02207)
Supplement: Supplementary file 1 — se3c02207_si_001.pdf [file se3c02207_si_001.pdf]

Supporting Information

Cluster-enhanced nanopore sensing of ovarian cancer marker peptides in urine

Thomas W. Rockett,[1] Mohammed Almahyawi,[1,2] Madhav L. Ghimire,[1] Aashna Jonnalagadda,[3] Victoria Tagliaferro,[3] Sarah J. Seashols-Williams,[4] Massimo F. Bertino,[1] Gregory A. Caputo,[3] and Joseph E. Reiner[1*]

1. Department of Physics, Virginia Commonwealth University, Richmond, VA 23284
2. King Fahd Medical Research Center, King Abdulaziz University, Jeddah 21589, Saudi Arabia
3. Department of Chemistry & Biochemistry, Rowan University, Glassboro, NJ 08028
4. Department of Forensic Sciences, Virginia Commonwealth University, Richmond, VA 23284

[*]Corresponding author: Joseph Reiner – jereiner@vcu.edu

Table of Contents *Page*

1. Summary of peptides studied S2
2. Low-frequency peptide fluctuation analysis S4
3. Detailed descriptions of data analysis S5
4. Peptide CD and fluorescence spectral analysis S7
5. Current traces for P16C1 and P16C15 S8

1. SUMMARY OF PEPTIDES STUDIED

Table 1: A summary of the peptides studied with corresponding physicochemical properties. Each peptide presents uniquely in the urine of ovarian cancer patients. Peptide naming scheme utilizes P*x*C*y* where *x* is the number of amino acid residues in the sequence and *y* indicates where in the sequence the cysteine residue is located. The last 5 peptides with the "F" letter correspond to fragments of some of the aforementioned peptides. The "FC" corresponds to a fragment resulting from chymotrypsin cleavage and "FT" results from trypsin cleavage. For example, P9C9FC corresponds to the peptide fragment that results from P9C9 undergoing cleavage with a chymotrypsin enzyme. The cysteine residue is highlighted in red for each peptide.

Peptide Name	Sequence	Length	Mass (g/mol)	Charge (pH 8)
P8C5	AVYYCQQY	8	1037.15	-0.1
P9C6	NNWDVCADM	9	1067.16	-2.1
P9C9	GLFEDTNLC	9	1011.11	-2.1
P13C1	CLPSKDYAEVGRV	13	1436.6	0
P16C1	CLPSKDYAEVGRVGYV	16	1755.99	-0.1
P16C4	VLRCEGPIPDVTFELL	16	1801.1	-2.1
P16C9	SLLEHRLECGANDMKV	16	1815.09	-1
P16C12	RDDLKKLLETECPQYI	16	1964.26	-1
P16C15	HEGSTVEKTVAPTECS	16	1674.79	-2
P17C2	SCQPPAEIPGYLPADTV	17	1757.96	-2.1
P19C2	SCQPPAEIPGYLPADTVHL	19	2008.26	-2
P20C2	WCFGPDGTGPNILTDITKGV	20	2091.37	-1
P23C1	CATSGDGLYEGLDWLSNQLRNQK	23	2568.8	-1
**FRAGMENTS**				
P9C6FC	DVCADM	6	652.7	-2
P9C9FC	EDTNLC	6	693.7	-2
P16C1FT	CLPSK	5	546.7	+0.9
P16C9FT	LECGANDMKV	10	1079.3	-1.1
P16C15FT	TVAPTECS	8	806.9	-1.1

Table 2: Origin protein name, length, location and nearby sequence for peptides listed in Table 1.

Peptide	Protein Name	Length	Location	Truncated Sequence (3 aa – PEPTIDE – 3 aa)
P8C5	Immunoglobulin kappa variable 3-20	116	105-112	EDF-(P8C5)-GSS
P9C6	Retinol binding protein 4	201	83-91	RLL-(P9C6)-VGT
P9C9	Histone H3.1	136	103-111	YLV-(P9C9)-AIH
P13C1	Haptoglobin isoform 2 preproprotein	347	207-219	MPI-(P13C1)-GYV
P16C1	Haptoglobin isoform 2 preproprotein	347	207-222	MPI-(P16C1)-SGW
P16C4	Alpha-1B-glycoprotein	495	419-435	RDA-(P16C4)-REG
P16C9	Tamm-Horsfall urinary glycoprotein	640	327-342	TDI-(P16C9)-SLG
P16C12	Protein S100-A8	93	31-46	AVY-(P16C12)-RKK
P16C15	Immunoglobulin lambda locus (IGL@)	235	217-235	QVT-(P16C15)
P17C2	Leucine-rich alpha-2-glycoprotein (LRG-1)	347	55-71	SSI-(P17C2)-HLA
P19C2	Leucine-rich alpha-2-glycoprotein (LRG-1)	347	55-73	SSI-(P19C2)-AVE
P20C2	Elongation factor 2	858	650-669	RKI-(P20C2)-QYL
P23C1	ADP-ribosylation factor 1	181	159-181	QAT-(P23C1)

2. LOW-FREQUENCY PEPTIDE FLUCTUATION ANALYSIS

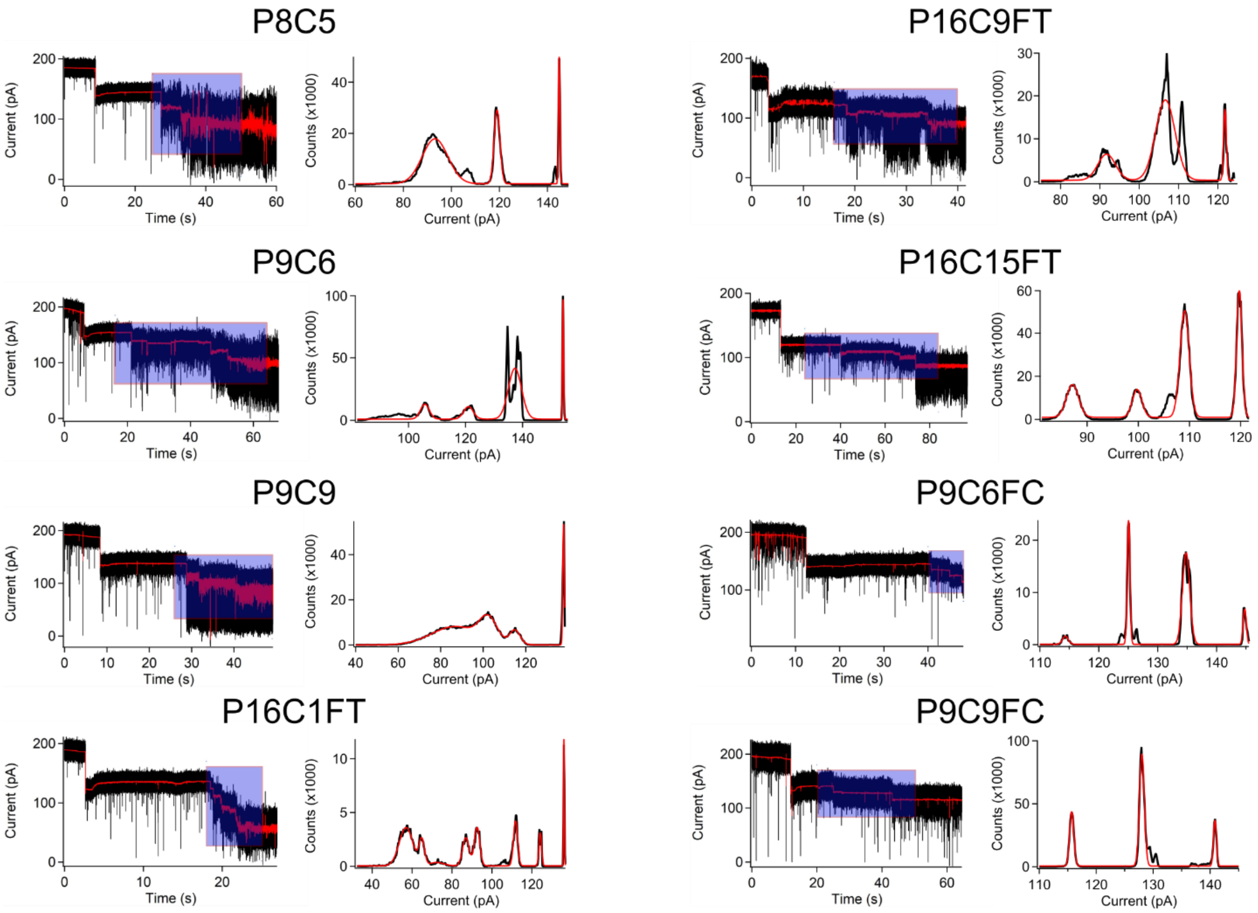

Figure S1: (Left) Sample current traces and corresponding all-points histograms for each of the low frequency peptides analyzed and reported in Figure 2 of the main article. The purple box highlights the region used to calculate the corresponding current histograms. (Right, black) All-points histograms for each of the low frequency peptides analyzed and reported in Figure 2 from the main article. (Right, red) Multicomponent Gaussian fits to the histograms.

3. DETAILED DESCRIPTIONS OF DATA ANALYIS

Open pore analysis: Open pore current blockades, like those shown in Fig. 3A of the main article, are analyzed with an in-house thresholding algorithm previously described (see reference 36 in the main article). The averaged open pore current, i_o, is calculated as the mean current 800 µs before and 800 µs after the onset of a blockade event. Blockade events are defined by current deviating by at least 5 standard deviations from the open pore current for at least 100 µs. The averaged blockade current, i, is the average current between the down and up steps of the blockade event. The blockade time, t_B, is the time between the up and down steps of the blockade event.

The overlap probability (OVL(a,b)) matrices reported in Figs. 3C, 4H and 5E in the main article show the probability of measuring a blockade from peptide type a and identifying it as peptide type b (e.g. the probability of measuring a blockade from P13C1 and identifying it as P16C1 as reported in Fig. 3C is 33.6%). To calculate the ab element of the OVL matrix we divide the blockade times and blockade depths into N^2 equally-sized regions (in linear scale) and then calculate the probabilities of finding peptide a and peptide b in each of these N^2 regions. We then sum the minimum probability in each region over all N^2 elements to calculate OVL(N). Finally, we calculate OVL(N) for N = 10, 11, ...,20 and report the average value in the matrix. The open pore data consists of 500 blockade events for each peptide, so we set an upper limit of N = 20 so that on average, each of the N^2 elements could contain at least 1 blockade event. We note that the diagonal components of the overlap matrix (OVL(a,a)) show the percentage of events for the a-type peptide that reside within the range of the graphs shown in Figs. 3B, 4G, 5D in the main article (e.g. 89.2% of the blockade events for P13C1 in Fig. 3B falls within $0 < i/i_o < 0.5$ and 0.1 ms $< t_B <$ 20 ms).

High frequency cluster-based analysis: Cluster-captured peptides reside in the pore for extended periods which enables accurate characterization and discrimination that is superior to open pore analysis. Rather than identify various moments from each current distribution (mean, variance, etc.), we instead compare the overall current distributions directly with chi-square analysis. Additionally, we utilize autocorrelation analysis to extract kinetic information, which can be used to improve detection selectivity.

We begin with the current distribution chi-square analysis. The current histograms in Fig. 3D and Fig. 4A-E in the main article are all calculated from 2-second current traces (the exception is P23C1 which were calculated from 20-second traces). To reduce event-by-event variability we shifted and normalized each current trace so the highest current state would correspond to 0 and the lowest current state would correspond to -1. To do this, we calculated a histogram from the unshifted/unnormalized current, then performed multipeak fitting with Igor Pro software (Igor Pro 6.37, Wavemetrics, Portland, OR) to identify the highest probability upper and lower current levels. We then subtracted the highest current level from the current trace and divided the lowest current level from this shifted current trace. This resulted in a current trace that fluctuated between 0 and -1 and we refer to this as our "normalized current", i_{norm}. An 1100 bin histogram ranging from i_{norm} = -1.4 to 0.66 with bin widths δi_{norm} = 0.001875 was calculated for each shifted and normalized current trace. From these histograms, we calculated the average and standard deviation of each peptide's histogram from the arithmetic mean of each of the individual distributions.

To find the probability that a type-a peptide would be identified as a type-b peptide by comparison of the histograms, we calculated the reduced chi-square value for type-a peptides using the following formula,

$$\chi^2_{r,(a,b)} = \frac{1}{N^*} \cdot \sum_{j=1}^{1100} \begin{pmatrix} \frac{\left(H_a\left(x_j\right) - M_b\left(x_j\right)\right)^2}{SD_b\left(x_j\right)^2} & \text{if } M_b\left(x_j\right) > M_{b,\text{threshold}} \\ 0 & \text{if } M_b\left(x_j\right) \le M_{b,\text{threshold}} \end{pmatrix} \tag{S1}$$

$$x_j = -1.4 + j\delta i_{\text{norm}}$$

where M_b and SD_b are the mean and standard deviation histogram values for the *b*-type peptide, and H_a is the histogram value of the *a*-type peptide. To reduce volatility in the calculation, we set a lower limit on the mean histogram value of $M_{b,\text{threshold}} = 50$ (10 for P16C4 and P16C15 peptides) so N^* is the number of bins in the average histogram where $M_b > M_{b,\text{threshold}}$.

Each *a*-type peptide yields a minimum of 5 different $\chi^2_{r,(a,b)}$ values from which we calculate an average and standard deviation for $\chi^2_{r,(a,b)}$. Assuming a normal distribution of $\chi^2_{r,(a,b)}$ values we calculated the probability that the measured $\chi^2_{r,(a,b)}$ value would be between 0 and 4 ($p = 0.05$). This yields, $P_{\chi^2}(a,b)$ the probability that the *a*-type peptide fluctuations would be interpreted as originating from a *b*-type peptide assuming a 95% probability of assigning an *a*-type fluctuation to an *a*-type peptide.

To incorporate the kinetic information we measured the normalized autocorrelation functions (ACF) for the same shifted and normalized current traces used for the chi-square analysis above. The normalized autocorrelation is defined by,

$$ACF(\tau) = \frac{\langle I(t) I(t+\tau)\rangle - \langle I\rangle^2}{\langle I^2\rangle - \langle I\rangle^2} \tag{S2}$$

where <*x*> corresponds to the time average of the parameter *x*. This definition assumes a stationary process where *t* is not relevant. Given the rate of fluctuations (~10 ms) versus the overall interrogation time (~10 s) this is an acceptable approximation. From single offset exponential fits to the normalized ACF we can extract the mean correlation time τ_{mean}. The number of events analyzed with autocorrelation analysis to extract the average and standard deviation of τ_{mean} for each peptide is as follows: P13C1 = 5, P16C1 = 7, P20C2 = 8, P23C1 = 5, P16C4 = 6, P16C9 = 7, P16C12 = 5, P16C15 = 7, P17C2 = 7, P19C2 = 7. Using these average and standard deviation values and assuming a normal distribution of mean correlation times, we calculated overlap probabilities, $P_\tau(a,b)$, for peptides of type *a* and *b*. Finally, assuming $P_{\chi^2}(a,b)$ and $P_\tau(a,b)$ are independent and uncorrelated probabilities, we calculate the overall probability of identifying a blockade from peptide *a* as peptide *b* with the following,

$$P(a,b) = P_{\chi^2}(a,b) P_\tau(a,b) \tag{S3}$$

This yields the matrix elements in Fig. 3F, 4F and 5E in the main article.

4. PEPTIDE CD AND FLUORESCENCE SPECTRAL ANALYSIS

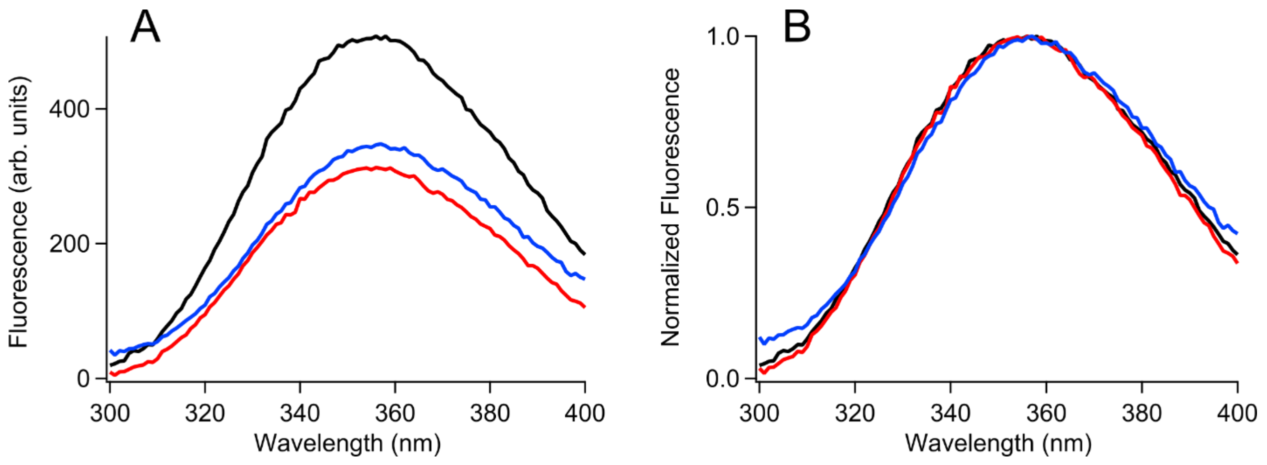

Figure S2: (A) Background subtracted and (B) intensity normalized tryptophan fluorescence spectra of P23C1 under varying conditions to explore buffer effects on peptide structure. Sample conditions are as follows: black = PBS only, red = PBS + 3 M KCl, and blue = PBS + 5 M Gdm-HCl. The near perfect agreement between all three normalized traces suggests that KCl and Gdm-HCl do not influence the emission, implying that the peptide behavior is unaffected in the high ionic strength solution used herein and that secondary structure of the free solution peptide is not observed. Peptide concentration and fluorescence excitation wavelength in all three samples is fixed at 2 μM and 280 nm respectively.

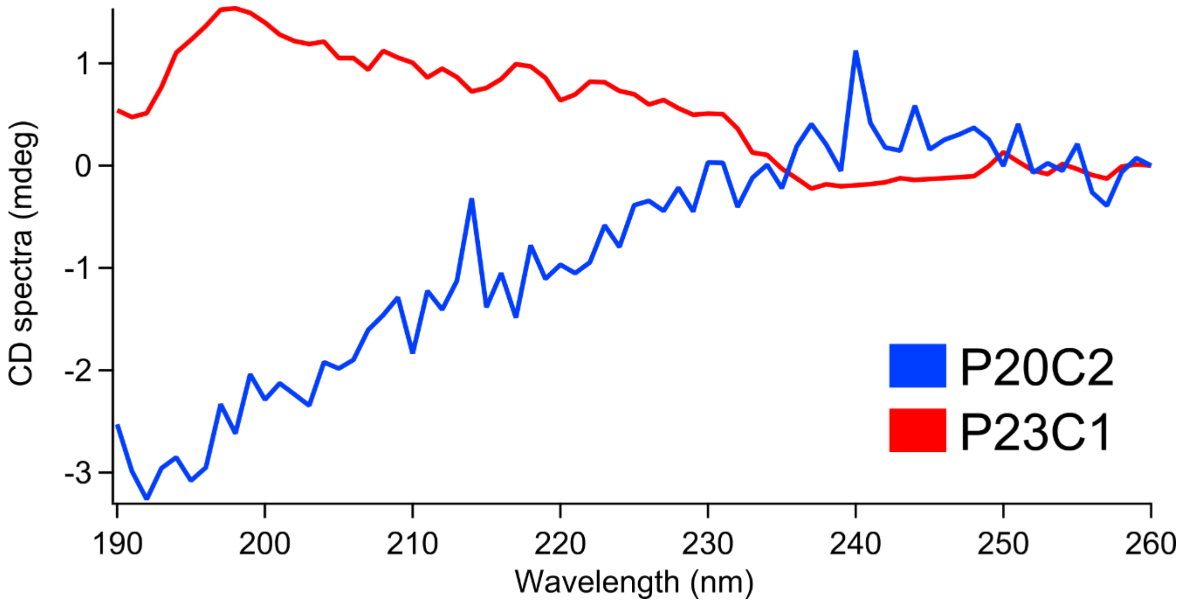

Figure S3: Circular Dichroism (CD) spectra of P20C2 and P23C1 peptides. The lack of a clear peak and the near zero values across the entire spectra suggests no secondary structure for these peptides in free solution. Background spectra from samples lacking peptide were subtracted. Data was collected in PBS buffer with peptide concentrations in both samples fixed at 20 μM. Both spectra show the average over 64 scans.

5. CURRENT TRACES FOR P16C1 AND P16C15

The normalized ACF for the P16C1 and P16C15 peptides reported in Fig. 4 of the main article show sizable variability between each peptide's ACF. This leads to larger differences in the correlation times and has a negative impact on the ability of the sensor to discriminate between different peptides. To better visualize this variance we present current traces that correspond to each of the autocorrelation functions for the two peptides. Figure S4 shows P16C1 traces and Fig. S5 shows P16C15 traces.

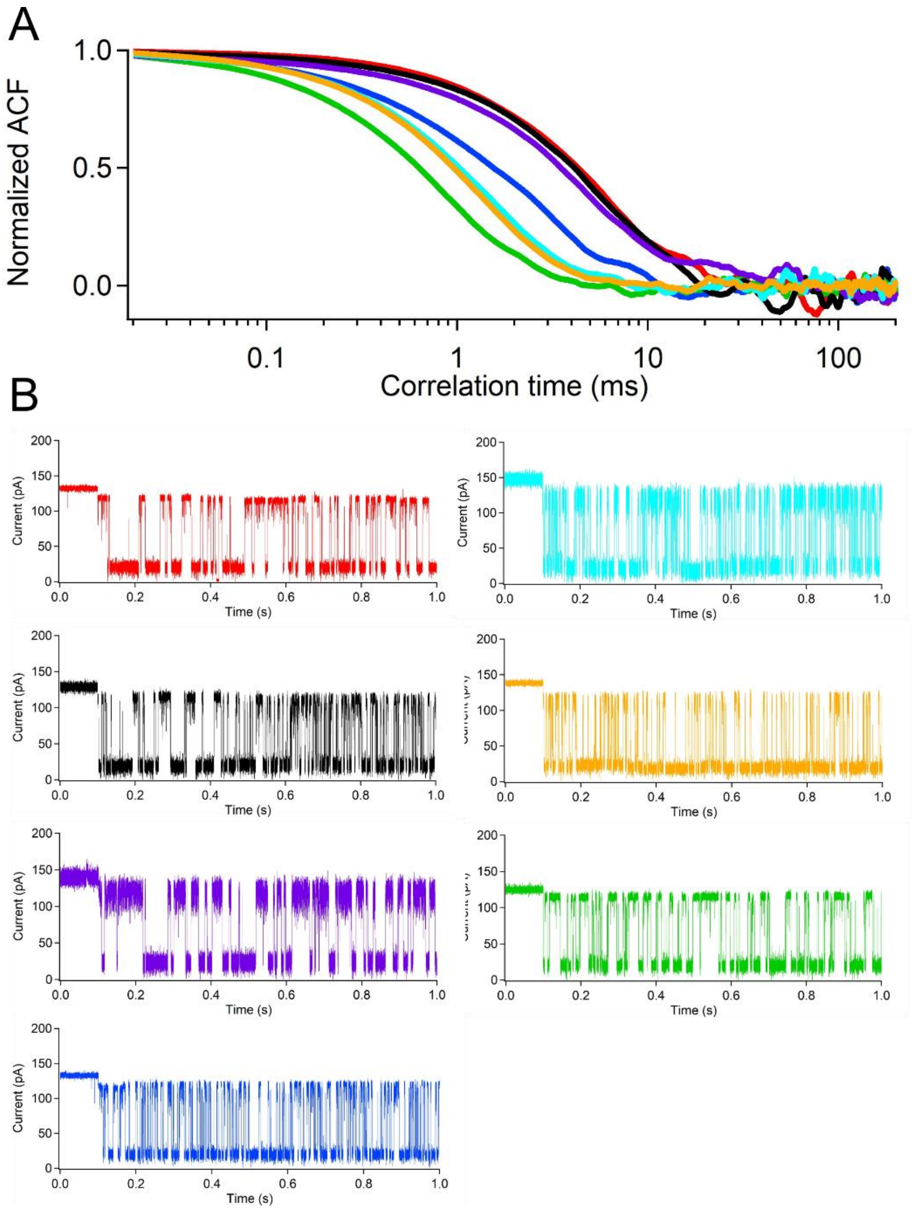

Figure S4: (A) Normalized autocorrelation curve for P16C1 reproduced from Fig. 4 of the main article. The mean correlation has been removed, but each curve has a unique color that corresponds to the current traces shown in (B). Current traces that show peptide capture at $t = 0.1$ s followed by 0.9 s of current fluctuations from a single captured peptide. All data collected under 70 mV applied transmembrane potential at pH 8.0 as described in the methods section.

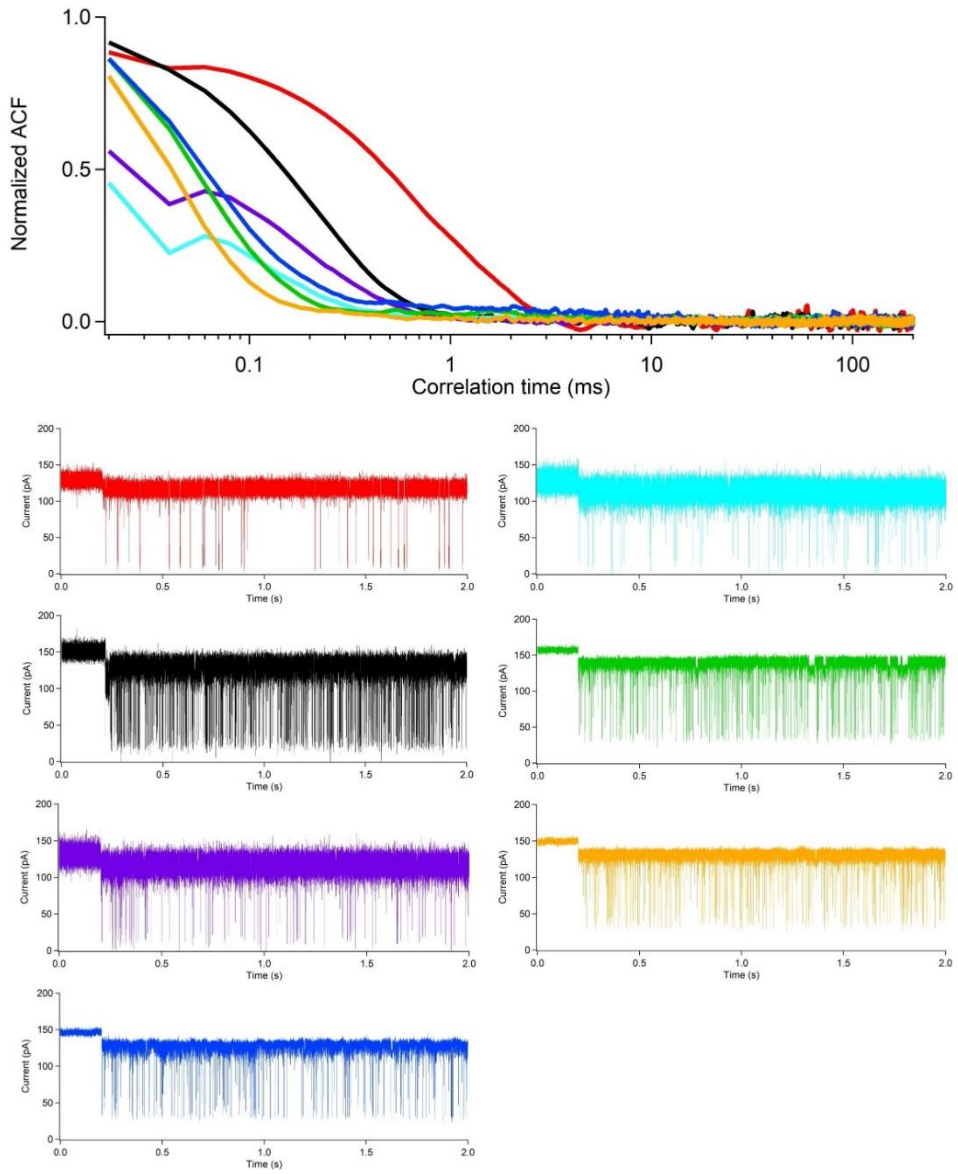

Figure S5: (A) Normalized autocorrelation curve for P16C15 reproduced from Fig. 4 of the main article. The mean correlation has been removed, but each curve has a unique color that corresponds to the current traces shown in (B). Current traces that show peptide capture at *t* = 0.2 s followed by 1.8 s of current fluctuations from a single captured peptide. All data collected under 70 mV applied transmembrane potential at pH 8.0 as described in the methods section.
